# Dataset on social and psychological effects of COVID-19 pandemic in Turkey

**DOI:** 10.1038/s41597-022-01563-4

**Published:** 2022-07-23

**Authors:** Emre Sari, Gamze Kağan, Buse Şencan Karakuş, Özgür Özdemir

**Affiliations:** 1 UiT the Arctic University of Norway, School of Business and Economics, Tromsø, 9037 Norway; 2 Uskudar University, Department of Occupational Health and Safety, Istanbul, 34662 Turkey; 3 Hacettepe University, Department of Psychology, Ankara, 06230 Turkey; 4 Istanbul University, Faculty of Law, Istanbul, 34116 Turkey

**Keywords:** Economics, Human behaviour, Government, Interdisciplinary studies, Society

## Abstract

This data was gathered to investigate how individuals’ levels of intolerance to distress and instant anxiety are related to some of the behaviors that people can change in response to the COVID-19 pandemic. We present a dataset based on a four-wave survey of the social and psychological effects of the COVID-19 pandemic in Turkey (N = 2,817). Turkey was heavily impacted by the first waves of infections in 2020, and citizens were forced to adapt to governmental measures. So, the dataset provides unique opportunities to investigate the COVID-19 pandemic’s role in shaping people’s intolerance to distress and instant anxiety. The survey considered personal cleaning behavior, bank/credit card usage, online spending habits, individual security perception, and stockpile behavior. Furthermore, in this data, whether an individual or a household member was officially diagnosed with COVID-19 and socio-demographic indicators were determined. Hence, the resulting dataset can enable various analyses on social, psychological, perceived security, and self-rated health, influencing how individuals’ levels of intolerance to distress and instant anxiety.

## Abstract (summary)

**Table:** 

Measurement(s)	Social Effects of Covid-19 Pandemic • Psychological Effects of Covid-19 Pandemic • State-Trait Anxiety Inventory • Measurement of Distress Intolerance • Economic Behavior • Usage of debit/credit card • Online Shopping • Stockpiling • Safety perception • Community safety perception • Feeling uneasy in crowded places • Self-rated health • Personal hygiene behaviour • Having a chronic disease • Diagnosed Covid-19 • Working Home office • Demographic
Technology Type(s)	Survey
Sample Characteristic - Organism	Homo sapiens
Sample Characteristic - Environment	Nomenclature of Territorial Units for Statistics (NUTS) of Turkey
Sample Characteristic - Location	Turkey

## Background & Summary

The COVID-19 pandemic ravaged the world began in Wuhan, China, in December 2019 and reached Turkey on March 10, 2020^1^. Medical research is still being conducted to determine the impact of this pandemic on humans in Turkey and around the world. This subject is receiving much attention in the social sciences (e.g., Aker and Mıdık^1^; Ferreira *et al*.^2^; Garbe *et al*.^3^; Karaar and Canli^4^; Leslie *et al*.^5^), as well as medical research. Like Mondino *et al*.^6,7^ stated that understanding how people perceive multiple risks and how major crises shape individual behaviors is required for ‘detecting windows of opportunity for policy change’^8,9^, ‘improving risk management strategies’^7,10^, and ‘supporting communication between decision-makers and the general public’^11^. Researchers can use this dataset to investigate how the COVID-19 pandemic has affected people’s emotions and behavior.

This paper presents a new dataset that provides unique opportunities to investigate the COVID-19 pandemic’s role in shaping people’s intolerance to distress and instant anxiety. We explore the public reflection of the epidemic in Turkey. A total of 2,817 people were surveyed online, and the results are compiled in this dataset. Data were collected from adults aged 18 to 65 and older, with an extensive demographic section covering location (province and rural-urban divide), income, employment status, occupation, occupational sector, family background, whether having a child, marital status, and gender identity. This data will allow researchers to investigate how the pandemic affects people differently depending on their age, economic impact, social status, and risk status. The survey remained online during those times. By timestamping, this data can be merged with other datasets: number of vaccinations, tests performed and positivity, hospital and ICU admissions, confirmed cases, confirmed deaths, policy responses, and other relevant variables. Furthermore, this data will be helpful for international comparative studies.

The epidemic has had a significant impact on the physical and mental well-being of many Turkish people. Despite the government’s efforts to keep COVID-19 under control (for details, see Table 1), the social isolation caused by the outbreak has had a significant impact on people’s lives. Due to the nature of the fight against COVID-19, these findings are not surprising in individuals who experience an increasing number of cases, new deaths, economic and other direct stress factors related to the pandemic^12^. To put it in a medical context, according to Koca^13^, people can readily access healthcare services in this country and obtain modern medical treatment. He notes that every patient in need of medical attention is admitted to a hospital, where they get specialized treatment, including intensive care and mechanical ventilation, if necessary.

**Table 1:** Precautions and significant events from the start of the COVID-19 pandemic until the end of April 2020 in Turkey.

Date	Measures implemented and significant events
Jan 10	The Coronavirus Scientific Committee (KvBK) was established within the Ministry of Health (MoH).
Jan 14	KvBK has published the first guide^36^ on testing and monitoring for healthcare professionals.
Jan 16	The Central Bank implemented the first economic action, and 75 basis points reduced the policy rate to 11.25%0^37^.
Jan 24	Thermal cameras were installed in all airports, and passengers arriving from China were screened^38,39^
Feb 7	The MoH has published a series of videos on social media to inform the public about COVID-19^40–43^
Feb 19	The Central Bank lowered the policy rate by 50 basis points to 10.75%^44^.
Mar 11	The first case of COVID-19 was detected^45^.
Mar 17	The first death from COVID-19 occurred^46^.
Mar 17	The Central Bank lowered the policy rate by 100 basis points to 9.75%^47^.
Mar 19	All sporting events across the country have been suspended, and public gathering places have also been temporarily closed. Restaurants were obliged to put Table 1 meter apart. Places of worship were closed; cultural, scientific, or artistic meetings were canceled^48^.
Mar 21	A partial curfew has been imposed for citizens over the age of 65 and with chronic diseases, and they are only allowed to leave their homes and walk-in open areas such as parks and gardens^49^.
Mar 22	Flexible working arrangements have been made for those working in public institutions and organizations^50^.
Apr 3	The Ministry of Interior (MoI) declared that all land, air, and sea entries and exits from 30 metropolitan provinces and Zonguldak province borders would be temporarily closed. He declared that citizens under the age of 20 (with exceptions) were prohibited from going out on the streets for a while, including 81 provinces. Furthermore, the Province Pandemic Boards were announced as having the authority to take the necessary additional measures^51^.
Apr 10	Interior Minister Süleyman Soylu announced the curfew in 30 provinces with metropolitan status and Zonguldak^52^.
Apr 17	MoI has banned all citizens from going out on the weekends for 30 provinces with metropolitan status and Zonguldak^53^.
Apr 21	With a circular issued by MoI, the curfews to be implemented on 23-24-25-26 April were announced^54^.
Apr 21	The Central Bank lowered the policy rate by another 100 basis points to 8.75%^55^.

These events occurred before the active collection of data and are critical for understanding individual mental development.

## Methods

This dataset is collected for quantitative research. In this data, we used the survey technique, which is a common quantitative research method. According to McKay (2005), survey research is the most controlled and structured method between experimental statistical research and qualitative research because it can use both statistical and qualitative analysis. The questionnaire to be used as a data collection method was divided into three sections and contained 55 questions.

**Fig. 1:**
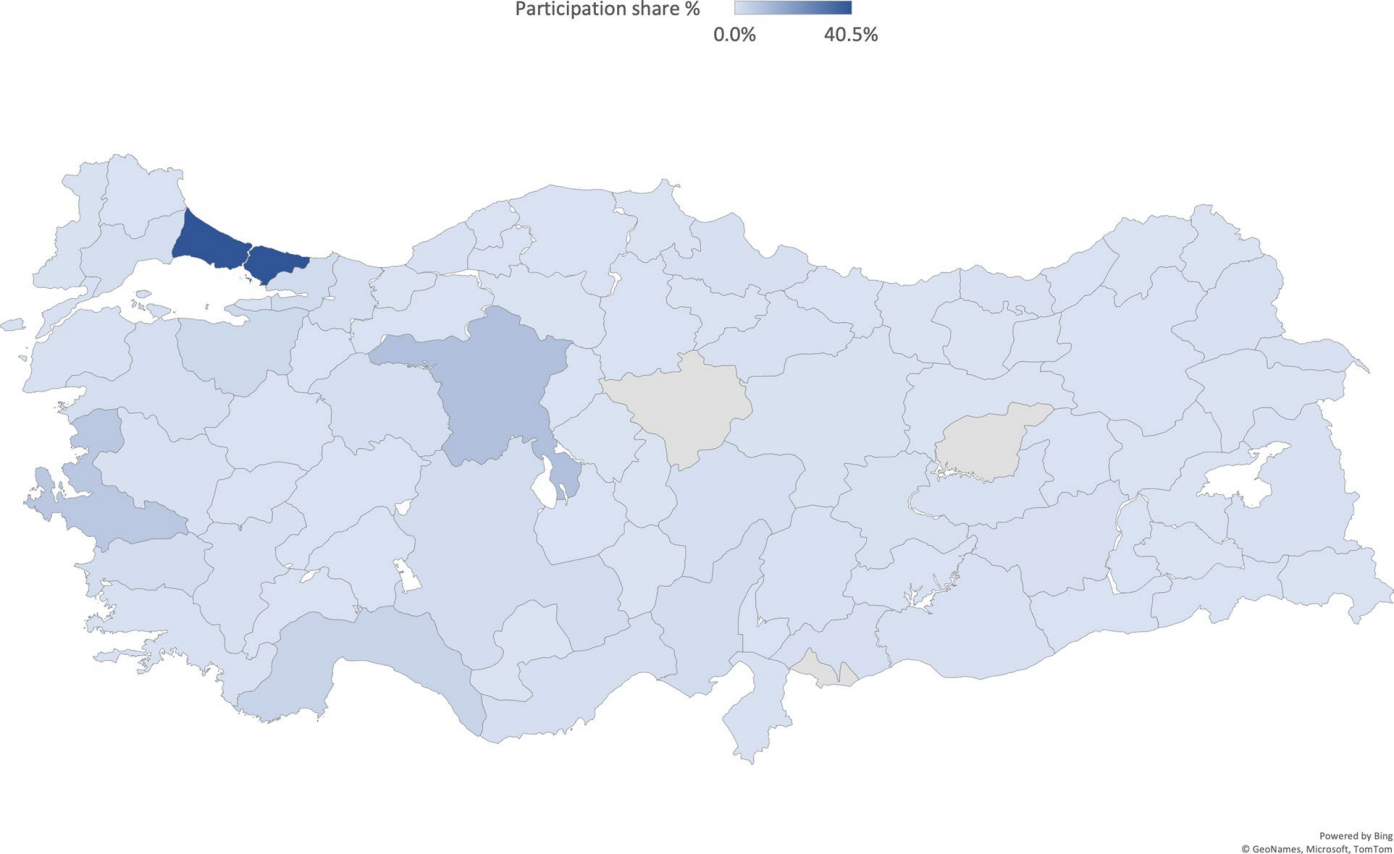
Spatial distribution of participants in Turkey. The number of people collected in each province is as follows: Istanbul, n = 1,155 (40.5%); Ankara, n = 281 (9.8%); Izmir, n = 219 (7.7%); Antalya, n = 122 (4.3%); other provinces, n = 1,076 (37,7%).

A total of 2,817 individuals participated in our study (65.3% women, mean age = 28.55 ± 10.4 years, range = 18–65 years and above). Participation in the survey was possible from all provinces in Turkey, and the participation rate is displayed on the map in Fig. 1. The survey was conducted online between April 13, 2020, and November 25, 2020. The survey was designed to assess the psychological impact of the social and psychological effects of the COVID-19 on participants. The total of the data obtained covers the adult population of Turkey with a 2% margin of error, a 50% response distribution, and a 95% confidence level. When we consider the study waves separately, the first two waves have 3%, the third wave 4%, and the fourth wave with a 7% margin of error and a 50% response distribution with a 95% confidence level (see Table 2). Only those under the age of 18 were excluded.

**Table 2:** Population of Turkey and sample sizes of survey waves.

	Population	Sample size	Margin of error	Confidence level
Total population of Turkey	80,810,525			
Above 15-year-old population	62,100,651			
**Total survey participation**		**2,817**	**2%**	**95%**
**Survey waves**				
Wave 1: *April 13–26*		1,124	3%	95%
Wave 2: *May 6–23*		975	3%	95%
Wave 3: *July 20 - August 8*		515	4%	95%
Wave 4: *November 14–25*		203	7%	95%

The numbers for the population of Turkey are taken from TurkStat, and they are Address Based Population Registration System Results in 2017^56^.

We asked 15-item demographic information section in the first section to determine the participants’ personal information, and the second section begins with health and economic behavior-related questions (see Table 3 for the questionnaire). The majority of participants (86%) responded comprehensively to all the questions.

**Table 3:** Items and observations about demographic, health, security, economic behavior, State-Trait Anxiety Inventory, and Measurement of Distress Intolerance.

Category	Variables	N	Missing	Distinct
Demographic	Age	2817	0	49
	Marital status	2817	0	4
	Gender	2817	0	3
	Age range of your child(ren) (if any)	2817	0	25
	City where you live (provinces)	2817	0	78
	Urban-rural divide	2817	0	2
	Education level	2817	0	7
	Mother’s education level	2773	44	8
	Employment status	2817	0	9
	Occupational sector	2817	0	7
	Occupation (ISCO-08)	2506	311	10
	Parent’s occupation (ISCO-08)	2817	0	10
	Home office	2411	406	3
	Current income (in month)	2817	0	6
	Last year income (average)	2817	0	6
Health	Self-rated health	2817	0	5
	Personal hygiene behaviour	2817	0	5
	Having a chronic disease	2817	0	2
	Diagnosed COVID-19	2817	0	2
Security	Safety perception	2817	0	5
	Community safety perception	2817	0	5
	Feeling uneasy in crowded places	2817	0	2
EconomicBehavior	Usage of debit/credit card	2817	0	5
	Online Shopping	2817	0	5
	Stockpiling	2817	0	2
State-Trait Anxiety Inventory (20-item sub-dimension)		2817	0	5
Measurement of Distress Intolerance		2817	0	5

The second part of the questionnaire includes the State-Trait Anxiety Inventory, developed by Spielberger *et al*.^14^. This scale measures anxiety symptoms in two sub-dimensions, state, and trait. The scoring of this scale, which consists of 40 items, is done on a 4-point Likert-type scale. High scores from the scale indicate a high level of anxiety. Within the scope of this research, the state anxiety sub-dimension of the inventory was used to measure the current anxiety of individuals. We used the 20-item part of this sub-dimension in our survey.

The third part of the questionnaire includes the Measurement of Distress Intolerance. This scale was developed to evaluate the individual’s perceived competence for resilience to various internal or external distresses and his behaviors towards coping^15^. This scale consists of 10 items and has a single factor structure. Items are scored on a 5-point Likert-type scale. High scores indicate higher intolerance to distress.

**Fig. 2:**
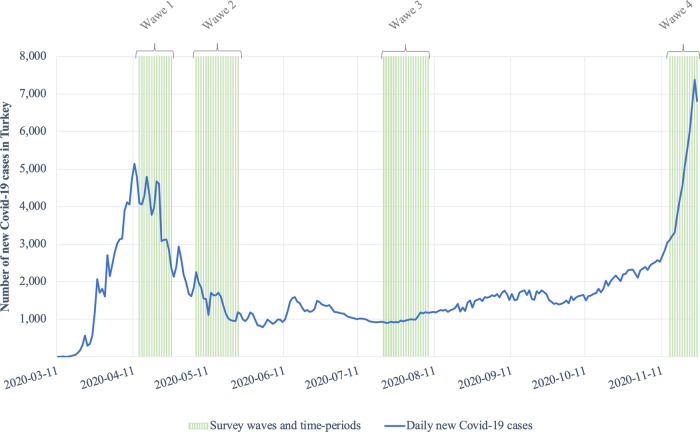
Number of new COVID-19 cases in Turkey and the data collection periods. We began gathering data on April 13th, 2020, and finished on November 25th, 2020. We announced the survey through social media accounts on April 18th, May 9th, July 20th, and November 16th of 2020. Daily new COVID-19 cases data obtained from Dong *et al*.^57^.

We conducted our survey online in four waves. We announced the first wave in the early period when the daily number of newly confirmed COVID-19 cases peaked (13–26 April 2020; N = 1,124); the second is the first period in which the number of COVID-19 cases decreased (6–23 May 2020; N = 958); the third is the end of the summer season when the number of cases is low (20 July – 8 August 2020; N = 513); the fourth is the period when the number of cases increased very rapidly (14–25 November 2020; N = 201) (see Fig. 2). The dataset also includes 36 people who completed the survey randomly from among the waves.

### Recruitment methods

We recruited participants primarily through social media posts (Instagram, Twitter, Facebook, and LinkedIn). In addition, we sent emails and messages directly to people who were in close contact with the researchers, asking them to share the survey with their networks. At the outset of the survey, we disclosed in the participant information part that no monetary or material compensation would be provided or requested in exchange for their participation. The questionnaire was hosted on the Google survey platform, Google Forms, and the survey questions took about five minutes to complete.

### Ethical approval

The research protocol for this study was approved by the Non-Invasive Research Ethics Committee of Uskudar University (nr: 61351342/2020-236), as well as the Ministry of Health of the Republic of Turkey. Each and every procedure used in the study was in accordance with the ethical standards established by the European Union (EU General Data Protection Regulation and FAIR Data Management). Additionally, we signed the World Medical Association’s Declaration of Helsinki: Ethical Principles for Medical Research Involving Human Subjects, which outlines ethical principles for medical research involving human subjects. Participants were informed that participation was completely voluntary and that the results would be kept confidential. The research protocol excluded the collection of data that was sensitive to privacy or that contained personally identifiable information (PII). All the participants in the study gave their informed consent after being briefed on the study’s objectives.

## Data Records

On the Mendeley Data platform, you can download data records in CSV format and files containing the questionnaires in both Turkish and English translations^16^. There is also an abbreviation guide for variable names included in the XLSX file as well. All of these resources can be found at the link provided: 10.17632/sv95c7ydpy.

## Technical Validation

Before asking participants to answer any questions, we presented an introductory page explaining the purpose of the study, the specifics of what participation would entail (including the identity and affiliations of the researchers, see *the online version* https://forms.gle/HFyqjhwhEcuPDZJW7), and confirmation that the research had received ethical approval from a legitimate review board and ethics committee^17^. Furthermore, we stated that they are free to withdraw from the study at any time and that we will not compensate you in any way, nor will any financial or material contribution be requested from you in exchange for your participation in the study.

Our survey used three main questionnaires: demographic, the State-Trait Anxiety Inventory, and the Measurement of Distress Intolerance. Spielberger *et al*.^14^ developed the State-Trait Anxiety Inventory. Its Turkish adaptation was made by Öner and Le Compte^18^. They found the internal consistency of the scale to be between 0.94 and 0.96 for the trait anxiety dimension and between 0.83 and 0.87 for the state anxiety dimension. The Measurement of Distress Intolerance scale consists of 10 items which are scored on a 5-point Likert-type scale. Çakır^19^ conducted the Turkish validity-reliability study of the scale, and the internal consistency coefficient was found to be 0.92.

We have prior experience in survey design for stress and anxiety, as well as demography research^20–25^. Additionally, we based the demographic, health and safety questions in the survey on previous research^8,26–33^, so that the questions appear reasonable in terms of obtaining the necessary data and comparable with previous findings. In the same way as Mondino *et al*.^6,7^, we administered the preliminary survey to a total of 19 people with various educational backgrounds. Among other things, we inquired whether the questions were straightforward and how they interpreted them. This step ensured that the responses to the questions were consistent with what we expected. It is worth to mention that the potential for human error in data entry is limited since the online survey system automatically collects responses.

## Usage Notes

This dataset provides excellent opportunities to investigate various aspects of psychology, economic behavior, and risk perceptions during natural hazards. It primarily provides detailed information from COVID-19 on individuals’ levels of anxiety and distress intolerance. Additionally, it includes information on economic behaviors such as stockpiling, online shopping, and bank/credit card use. It also provides data on people’s perceptions of personal safety and public trust in the national government. Finally, it aids researchers in observing changes in the process by addressing the nature of the various responses to the COVID-19 pandemic’s different waves and the context of the pandemic’s multiple stages.

Indeed, this dataset can be enhanced significantly by adding other relevant and important datasets for international comparative studies, such as Yamada *et al*.’s^34^ COVIDiSTRESS Global Survey on the psychological and behavioral effects of the COVID-19 and Mondino *et al*.’s^6^ COVID-19 public impressions in Italy and Sweden datasets (for more details, see *Supplementary Information*). As Mondino *et al*.’s^6^ stated, we want to emphasize that such sensitive knowledge is scientifically valuable and can guide policymakers in developing and updating risk management policies during natural hazards.

The dataset is accompanied by survey questionaries files in Turkish and English in PDF format that includes variable descriptions, and R-codes for data processing are publicly available on the Mendeley Data platform^16^. There is no need to request access to download data for academic purposes. Semicolons separate columns in the CSV file.

### Limitations

Researchers should consider the sample’s skewness for respondents, such as being female, single, well-educated, and residing in Istanbul. Therefore, the dataset’s samples are not representative of the Turkish population, particularly gender, education level, and marital status. As a result, researchers who must address this issue can weigh the data by referring to publicly available demographic data for Turkey and applying suitable weights to the variables (e.g., https://data.tuik.gov.tr/)^35^. Users of this dataset should also be aware that these reports are based on self-report and subjective evaluations of participants. We have no attempt or chance to verify the accuracy of the participants’ answers externally.

## Supplementary information

Supplementary Information

## Data Availability

Raw data, as well as R-code for cleaning, are freely available at: 10.17632/sv95c7ydpy^16^.
