# Supplementary material for: Dataset on social and psychological effects of COVID-19 pandemic in Turkey
Source: Sci Data. 2022 Jul 23;9:441. doi: 10.1038/s41597-022-01563-4 (PMC9308400; doi:10.1038/s41597-022-01563-4)
Supplement: Supplementary file 1 — Supplementary Information [file 41597_2022_1563_MOESM1_ESM.pdf]

Dataset on social and psychological effects of COVID-19 pandemic in Turkey

- Supplementary Information -

Here are some examples of data sources you might consider combining or comparing for your study:

1. Turkish Statistical Institute (TUIK)
   Population and demography
   Employment, unemployment, and wages
   Health and social protection
   Income, living, consumption and poverty
   Etc.
   https://data.tuik.gov.tr/

2. Geographic Statistics Portal for Turkey
   https://cip.tuik.gov.tr/

3. Coronavirus Pandemic (COVID-19)
   https://ourworldindata.org/coronavirus

4. COVID-19 European regional tracker
   https://doi.org/10.1038/s41597-021-00950-7

5. A cross-country database of COVID-19 testing
   https://doi.org/10.1038/s41597-020-00688-8

6. COVID-19 outbreak response, a dataset to assess mobility changes in Italy following national lockdown
   https://doi.org/10.1038/s41597-020-00575-2

7. A real-time survey on the psychological impact of mild lockdown for COVID-19 in the Japanese population
   https://doi.org/10.1038/s41597-020-00714-9

8. Public perceptions of multiple risks during the COVID-19 pandemic in Italy and Sweden
   https://doi.org/10.1038/s41597-020-00778-7

9. COVIDiSTRESS Global Survey dataset on psychological and behavioural consequences of the COVID-19 outbreak
   https://doi.org/10.1038/s41597-020-00784-9

10. Anxiety and Depression
    https://www.cdc.gov/nchs/covid19/pulse/mental-health.htm

11. Survey data on the consequences of COVID-19 and home confinement on the educational community and families in Spain
    https://doi.org/10.1016/j.dib.2021.107606

12. The psychological and social impact of COVID-19 (UK)

https://www.ukri.org/news-and-events/tackling-the-impact-of-covid-19/researching-the-impact-of-coronavirus/the-psychological-and-social-impact-of-covid-19/

13. Impact of COVID-19 on Mental Health: A Longitudinal Study Using Penalized Logistic Regression
https://doi.org/10.1101/2021.02.21.21252159

14. Perceptions and opinions on the COVID-19 pandemic in Flanders, Belgium: Data from a five-wave longitudinal study
https://doi.org/10.17632/mhx3p7w3d6.9

15. Apple - Mobility Trends
https://covid19.apple.com/mobility

16. Google - Community Mobility Reports
https://www.google.com/covid19/mobility/

17. 2020 HRS COVID-19 Project
https://hrsdata.isr.umich.edu/data-products/2020-hrs-covid-19-project
